# Eco-friendly textile desizing with indigenously produced amylase from *Bacillus cereus* AS2

**DOI:** 10.1038/s41598-023-38956-3

**Published:** 2023-07-25

**Authors:** Aneela Rehman, Asma Saeed, Wajeeha Asad, Ibrar Khan, Azam Hayat, Mujaddad Ur Rehman, Tawaf Ali Shah, Baye Sitotaw, Turki M. Dawoud, Mohammed Bourhia

**Affiliations:** 1 Department of Microbiology, Abbottabad University of Science & Technology, Havelian, 22010 Pakistan; 2 Department of Microbiology, University of Karachi, Karachi, Pakistan; 3 Shandong Research Centre of Engineering and Technology for Clean Energy, Shandong University of Technology, Zibo, China; 4 Department of Biology, Bahir Dar University, P.O. Box 79, Bahir Dar, Ethiopia; 5 Department of Botany and Microbiology, College of Science, King Saud University, P.O. Box 2455, Riyadh, 11451 Saudi Arabia; 6 Department of Chemistry and Biochemistry, Faculty of Medicine and Pharmacy, Ibn Zohr University, 70000 Laayoune, Morocco

**Keywords:** Biotechnology, Microbiology

## Abstract

Starch is added to the fabric surface to secure weaving process. During finishing these sized particles are removed from the fabric and prepared it for printing and dyeing. Chemicals de-sizing agents damage fabric surfaces and reduce the quality of the product. An alternative to these conventional desizing agents is the use of biological molecules i.e. enzymes. The current study compares traditional de-sizing to bio-based de-sizing methods, as well as the optimization of fabric desizing settings using crude amylase. Amylase-producing *Bacillus cereus* AS2 was isolated from indigenous soil samples. The maximal fermentative de-sizing capability was discovered at 72 h, with no fabric surface degradation. Chemical desizing showed that the fabric lost all sizing agents to TEGEWA scale 9 within 1 h in presence of 5N HCl. Optimal studies for desizing showed that 1000 IU/ml of amylase resulted in maximum de-sizing within 15 h at 60 °C and 0.5% Triton-X. Water absorbance and weight loss, both parameters were used to check the desizing efficacy and it was found that de-sizing to same scale was occurred in the case of enzyme as well as commercially desized fabric. Enzyme desized cloth was found to be free of any starch particles in SEM micrographs, identical to industrially de-sized fabric, ensuring bioprocess efficacy.

## Introduction

After cellulose, starch is the second major reservoir of carbohydrates. It is stored in cereals, tubers, and leaves of plants. It is not only the major constituent of the human diet but also has importance in various industrial sectors. Starch is one of the most commonly used sizing agents that is made from natural raw ingredients. It is relatively inexpensive and readily available. Starch and its derivatives account for over 75% of all sizing agents used around the world. Conventionally starch sizes were usually removed by applying acids, but now microbial enzymes are gaining more interest as an alternative de-sizing agent^1^. Enzymes that hydrolyze starch are divided into four categories: endoamylases, exoamylases, de-branching enzymes, and transferases. Members of the endo amylase group cleave the internal 1, 4 bonds of amylose and amylopectin (α-amylase) subunits which result in the production of oligosaccharides. Exoamylases, the second group of starch hydrolyzing enzymes, target either 1, 4 (amyloglucosidase) or 1, 4 and 1, 6 (β-amylase). Exoamylases attack external glucose residues of amylose and amylopectin subunits and either yield only glucose (glucoamylase) or maltose and dextrin (β-amylase) as the end product. De-branching enzymes, a third group of enzymes, exclusively hydrolyze 1, 6 glycosidic bonds: pullulanase and isoamylase. Enzymes belonging to the fourth group, Transferases, cleaved 1, 4 glycosidic bonds (amylomaltase) and transfer part of the donor molecule to an acceptor (glycosidic) and form a new glycosidic bond.

Amylases can be isolated from all living systems including animals, plants and microorganisms^2,3^. Animals and plants as amylase sources are less important as enzymes from these organisms cannot fulfill industrial demands. For example, amylases from these sources are not much stable at high temperatures for many industrial processes. Therefore, microbial enzymes are the preferred for industrial needs^4^. Microbial amylases have thus various advantages over other sources such as cost-effectiveness, reliability, predictability, high stability, and eco-friendly behavior^5,6^. Many microbial species have been reported for amylases production such as *Aspergillus oryzae*, *Aspergillus fumigates*, *Hemicola lanuginose*, *Rhizopus Hemicola*, *Aspergillus stellate*, *Thermococcus hydrothermalis*, *Pyrococcus furiosus*,*Bacillus subtilis*, *Bacillus amyloliquifaciens*, *Bacillus cereus Bacillus stearothermophilus, Bacillus thuringiensis, Bacillus coagulans, Bacillus thermoleovorans* and *Bacillus licheniformis*^7^.

Among industrially important enzymes, amylases are important as these were the first to be produced at an industrial scale and commercialize for various purposes. These industrially important enzymes are used in various sectors. In the paper and pulp industry,amylase enzyme is used to modify starch molecules during paper coating process^8^. Similarly, amylase is used in fermenting agricultural crops’ sugar to produce bioethanol which is an alternative to crude oil. In the food sector amylase is used extensively in baking, brewing, digestive aids,cake production, and juice preparations^4^. Most importantly amylase is used in the detergent and textile industry to remove starch residues from fabric^9^.

De-sizing, souring and bleaching are preparatory stages for wet-processing treatments like dyeing, printing, and finishing on cotton fabrics^10^. The removal of adhesive compounds (sizes) from fabric threads that are applied during the weaving process to prevent yarn from damage and breakage is known as de-sizing. Chemicals such as alkalis, acids, or oxidizing agents often used to remove these sized particles from the fabric. However, these chemicals not only eliminate non-cellulosic contaminants but also they cause the cloth to lose weight and strength. Furthermore, these compounds cause wastewater to have high COD, BOD, and TDS levels. Textile finishing industries’ sizing agents result in effluent with a COD of 50–80 percent. Enzyme technologies, such as the usage of amylases, result in the breakdown of starch into dextrin and simpler glucose units, resulting in a more environmentally friendly production process^11^.

Nowadays attempts are made to replace fabric treatment processes by environment friendly enzyme-based techniques^12,13^. Mild reaction conditions during enzymatic processes make this technique more eco-friendly^12,14,15^. Many studies have shown that amylase is used commercially for de-zinging and sour purposes^16^. However, rather than being effective, bio-based approaches were plagued by issues such as removing black or dark brown seed coat pieces with or without connected linters and fibers^17^. These are the most difficult contaminants to eliminate, even when employing strong acid solutions, hence additional bleaching operations are required to achieve perfect cotton whiteness. Enzymes primarily hydrolyze microscopic seed-coat fibers that adhere to fiber fragments, allowing for the simple removal of leftover seed-coat fragments during the chemical bleaching process^18^.

It is critical to introduce current affordable enzyme preparations in bulk quantities for industrial deployment of any technology. As a result, *Bacillus cereus* AS2, which produces extracellular amylase, was isolated and enzyme production was enhanced using various parameters. The current research examines the use of amylase in fabric de-sizing and the optimization of several process parameters.

## Materials and methods

### Microorganism

The test organism in the current study was isolated from a garden soil sample by serial dilution method on Luria basal medium containing substrates. The selected isolate was subjected to microscopic, morphological, and molecular identification. Based on 16S rRNA gene sequence data, the organism was identified as *Bacillus cereus* (Accession number MK640654). The isolated organism was preserved on a basal medium at 37 °C for further studies^19^.

### Amylase production

Erlenmeyer flask containing 100 ml Luria basal media containing (g/l): starch 10, yeast extract 1.0, NaCl 8.9, and pH 7.0, and was inoculated with 8 percent seed culture and incubated at 37 °C for 24 h at 150 rpm shaker. After complete fermentation, the broth media was subjected to centrifugation at 8000*g* for 15 min. Cell-free supernatant was used to determine enzyme activity. Substrate (1% starch) solution was mixed in equal quantities with enzyme solution (100 μl). The reaction mixture was incubated at 50 °C for 15 min followed by the addition of 96 mM DNS (100 μl) to the mixture. The tube containing the reaction mixture was boiled for 15 min followed by cooling to room temperature at ice. Volume was adjusted by the addition of 900 μl distilled water. Enzyme activity was determined by comparing to the maltose standard curve by recording the absorbance at 540 nm. One unit of amylase activity is defined as one milligram of reducing sugar (maltose) produced from starch by an enzyme in 15 min at pH 7.0 and 50 °C^20^.

### Fermentative desizing

Fabric desizing was carried out using a growing medium containing 8% seed culture. At 40 °C, de-sizing potential and amylase production were assessed at various time intervals (0–120 h). Fabric samples were taken out at various time intervals. Desized fabric samples were washed at 100 °C for 5 min and then washed gently in cold water. Desizing efficacy was assessed using the TEGEWA rating system and SEM micrographs. To compare fabric de-sizing, a negative control (growing medium without inoculum) was used^21^.

### Conventional desizing

Different concentrations of HCl (0.5N to 5N) were tested for the conventional de-sizing process. After desizing, fabric samples were rinsed with soft water and then air-dried. TEGEWA rating was used to measure de-sizing efficiency. Post-de-sizing changes were visualized via SEM micrographs^21^.

### Optimization of fabric desizing

Desizing conditions were optimized for maximum fabric desizing using crude enzyme. These parameters include temperature (30–70 °C), time (1–24 h), enzyme concentration (200–1600 IU/ml) and wetting agents (Tween 80 and Triton X)^22^.

### Desizing efficacy test

Fabric desizing potency was evaluated by following tests procedures in Dalvi and Anthappan^23^

#### Starch detection

Desized fabric samples were washed for 10 min at 100 °C followed by cold washing and air drying. Samples were dipped in TEGEWA solution for 1 min. Fabric samples were then rinsed with distilled water and dabbed with filter paper. Starch content was checked by comparing it to TEGWA scale.

#### Percent weight loss

Weight loss of desized fabric was calculated by washing fabric and drying it for 1 h at a high temperature (105 °C). By using the following equation weight, the loss was calculated after complete drying.

$${\text{Weight}}\,{\text{ loss }}\left( \% \right) \, = \, Z1{ - } \, Z2 \, \times \, 100$$

where Z1 and Z2 indicate the weight of fabric before and after desizing, respectively.

#### Drop absorbency and tensile strength

The wettability of desized fabric was determined by AATCC Test Method 79-2007. At room temperature, a drop of distilled water from height of 1 cm was allowed to drop on the stretchable surface of the fabric. The time between contact of the water drop on the fabric surface and its disappearance was recorded. ASTM procedure D5035 (https://www.astm.org/Standards/D5035.html) was used to determine the tensile strength of fabric.

### TEGEWA test

**Figure 1:**
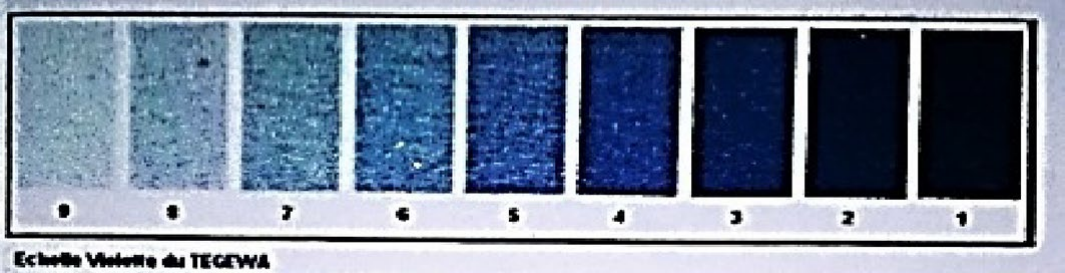
TEGWA scale.

Potassium iodide (10 g) and iodine crystals (1 g) were dissolved in distilled water (800 ml) by agitation and stirring. Ethanol (200 ml) was added to adjust the final volume up to 1 L. At room temperature, potassium iodide solution was spotted on fabric and gently dabbed with filter paper to remove extra amount. Violet scale readings were compared to assessed changes in fabric color (Fig. 1)^24^.

### Scanning electron microscopy (SEM)

SEM micrograph of biologically desized, chemically treated and controlled was recorded through JSM-6380A JEOL microscope (Japan Electron Optics Laboratory Co., Ltd., Tokyo, Japan)^21^.

## Results

### Screening for amylase production

Identified bacillus strain was subjected to qualitative and quantitative amylase screening. Enzyme index of 2.11 mm was observed on plate screening whereas fermentative screening showed 1200 IU/ml/min enzyme units after comparing with maltose standard curve.

### Fermentative desizing

**Figure 2:**
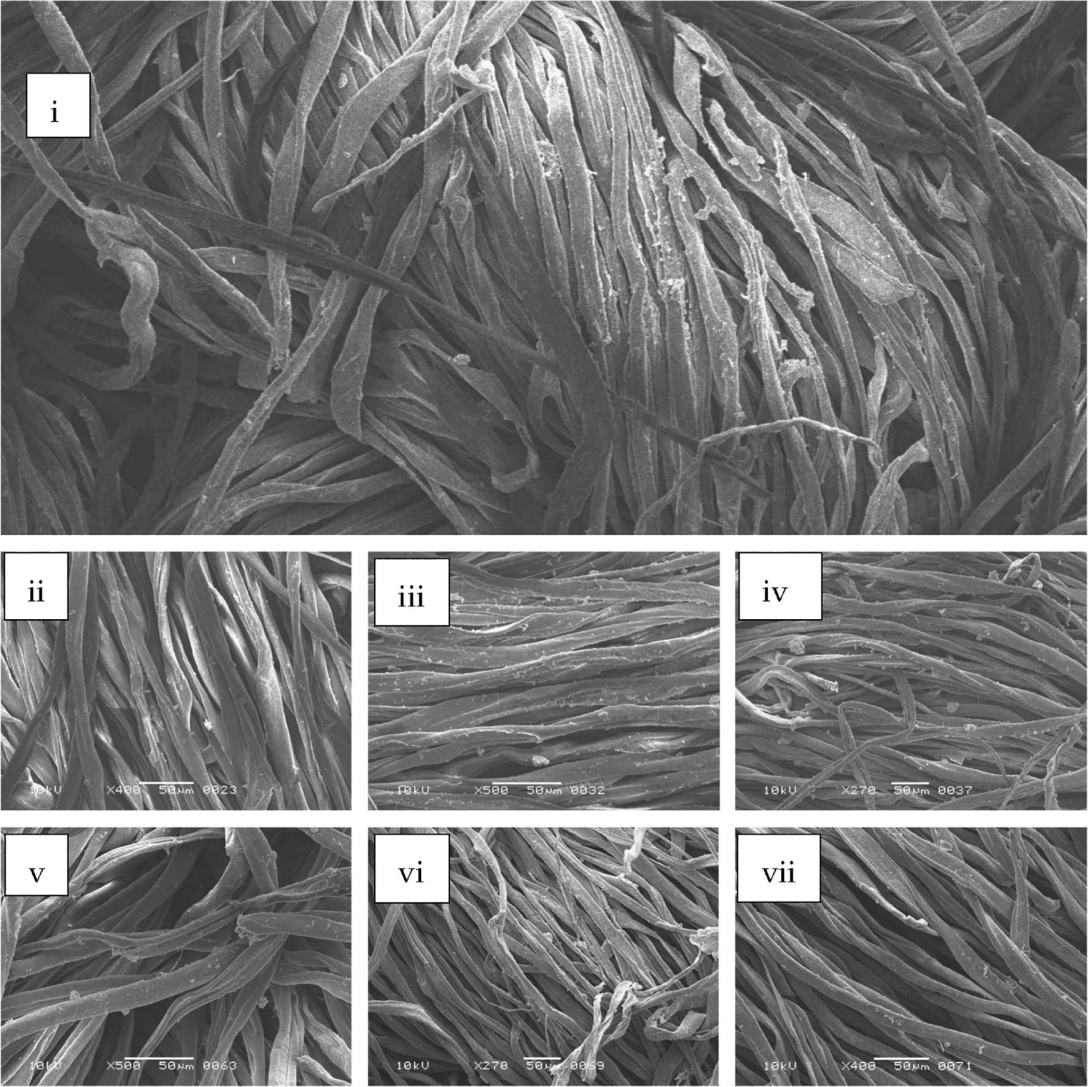
SEM results of fabric samples after enzymatic desizing. (**i**) 0 h, (**ii**) 5 h, (**iii**) 24 h, (**iv**) 48 h, (**v**) 72 h, (**vi**) 96 h, (**vii**) 120 h.

Fabric samples were incubated at different time intervals in fermentation medium. Desizing efficacy was determined by comparing it with the TEGWA rating (Table 1). It was observed that starch utilization starts at 5 h and remained continued until 72 h. After the optimum period gradual decrease in enzyme production was observed. SEM micrograph showed that no fabric damage occurred throughout the process (Fig. 2).

**Table 1:** Fabric desizing at different time period (TEGEWA rating).

Time (h)	0	5	24	48	72	96	120
TEGEWA Rating	2 ± 0	5 ± 0	9 ± 0	9 ± 0	9 ± 0	9 ± 0	9 ± 0

### Conventional desizing studies

Different concentrations of diluted HCl (0.5–5N) were also checked at various time intervals. TEGWA scale was used to compare the results of chemical de-sizing. Results indicated that maximum desizing (TEGEWA scale 9) occurred in 5N HCl within 1 h. Desizing at lower concentrations of HClL was not efficient for the time (Table 2).

**Table 2:** Fabric desizing in presence of HCl.

HCl concentration (N)	TEGEWA scale									
	Time (h)									
	1/2	1	2	3	6	10	14	18	22	24
0.5	2 ± 0	2 ± 0	2 ± 0	2 ± 0	2 ± 0	2 ± 0	2 ± 0	2 ± 0	2 ± 0	2 ± 0
1.0	2 ± 0	2 ± 0	2 ± 0	2 ± 0	2 ± 0	2 ± 0	2 ± 0	3 ± 0	3 ± 0	3 ± 0
1.5	2 ± 0	2 ± 0	2 ± 0	2 ± 0	2 ± 0	2 ± 0	3 ± 0	3 ± 0	4 ± 0	5 ± 0
2.0	2 ± 0	2 ± 0	2 ± 0	2 ± 0	3 ± 0	4 ± 0	4 ± 0	5 ± 0	5 ± 0	6 ± 0
2.5	2 ± 0	2 ± 0	2 ± 0	3 ± 0	3 ± 0	4 ± 0	4 ± 0	5 ± 0	6 ± 0	6 ± 0
3.0	2 ± 0	2 ± 0	3 ± 0	4 ± 0	4 ± 0	4 ± 0	4 ± 0	5 ± 0	6 ± 0	6 ± 0
3.5	2 ± 0	2 ± 0	3 ± 0	4 ± 0	4 ± 0	5 ± 0	5 ± 0	5 ± 0	6 ± 0	7 ± 0
4.0	2 ± 0	2 ± 0	2 ± 0	5 ± 0	5 ± 0	5 ± 0	5 ± 0	6 ± 0	6 ± 0	8 ± 0
4.5	5 ± 0	5 ± 0	6 ± 0	6 ± 0	7 ± 0	7 ± 0	8 ± 0	8 ± 0	8 ± 0	9 ± 0
5.0	8 ± 0	9 ± 0	9 ± 0	9 ± 0	9 ± 0	9 ± 0	9 ± 0	9 ± 0	9 ± 0	9 ± 0

### Fabric desizing using crude enzyme

#### Temperature and time

The crude enzyme was used to check desizing at different temperatures and time intervals. The effect of temperature and time on desizing showed that maximum starch from the fabric surface was removed at 60 °C within 15 h (Table 3).

**Table 3:** Temperature and time optimization for fabric desizing potential of amylase.

Time (hours)	Temperature (°C)								
	30	35	40	45	50	55	60	65	70
TEGWA rating									
0	2 ± 0	2 ± 0	2 ± 0	2 ± 0	2 ± 0	2 ± 0	2 ± 0	2 ± 0	2 ± 0
1	2 ± 0	2 ± 0	3 ± 0	3 ± 0	5 ± 0	6 ± 0	6 ± 0	6 ± 0	3 ± 0
2	2 ± 0	3 ± 0	3 ± 0	4 ± 0	5 ± 0	6 ± 0	7 ± 0	6 ± 0	3 ± 0
3	2 ± 0	3 ± 0	3 ± 0	4 ± 0	6 ± 0	7 ± 0	8 ± 0	6 ± 0	3 ± 0
4	2 ± 0	3 ± 0	4 ± 0	5 ± 0	6 ± 0	7 ± 0	8 ± 0	6 ± 0	3 ± 0
5	2 ± 0	3 ± 0	4 ± 0	5 ± 0	6 ± 0	7 ± 0	8 ± 0	7 ± 0	4 ± 0
6	2 ± 0	4 ± 0	4 ± 0	6 ± 0	7 ± 0	8 ± 0	8 ± 0	7 ± 0	4 ± 0
9	3 ± 0	4 ± 0	5 ± 0	6 ± 0	7 ± 0	8 ± 0	8 ± 0	7 ± 0	4 ± 0
15	3 ± 0	4 ± 0	5 ± 0	6 ± 0	7 ± 0	8 ± 0	9 ± 0	8 ± 0	4 ± 0
21	3 ± 0	5 ± 0	5 ± 0	6 ± 0	7 ± 0	9 ± 0	9 ± 0	8 ± 0	4 ± 0
27	3 ± 0	5 ± 0	6 ± 0	7 ± 0	8 ± 0	9 ± 0	9 ± 0	8 ± 0	4 ± 0

#### Enzyme concentrations

The combined effect of various enzyme concentrations and temperature on fabric desizing was checked for 1 h. At a temperature range of 55–60 °C, maximum desizing occurred in the presence of 1000 IU/ml of amylase. Further increase in enzyme concentration did not a affect desizing anymore (Table 4).

**Table 4:** Optimization of enzyme concentration for fabric desizing.

Enzyme concentration (IU/ml)	Temperature (°C)								
	30	35	40	45	50	55	60	65	70
200	2 ± 0	3 ± 0	5 ± 0	5 ± 0	5 ± 0	5 ± 0	6 ± 0	5 ± 0	3 ± 0
400	3 ± 0	3 ± 0	5 ± 0	5 ± 0	5 ± 0	6 ± 0	6 ± 0	5 ± 0	3 ± 0
600	3 ± 0	3 ± 0	5 ± 0	5 ± 0	6 ± 0	6 ± 0	7 ± 0	5 ± 0	3 ± 0
800	3 ± 0	3 ± 0	5 ± 0	5 ± 0	6 ± 0	6 ± 0	7 ± 0	5 ± 0	3 ± 0
1000	3 ± 0	3 ± 0	5 ± 0	5 ± 0	6 ± 0	6 ± 0	7 ± 0	5 ± 0	3 ± 0
1200	3 ± 0	3 ± 0	5 ± 0	5 ± 0	6 ± 0	6 ± 0	7 ± 0	5 ± 0	3 ± 0
1400	3 ± 0	3 ± 0	5 ± 0	5 ± 0	6 ± 0	6 ± 0	7 ± 0	5 ± 0	3 ± 0
1600	3 ± 0	3 ± 0	5 ± 0	5 ± 0	6 ± 0	6 ± 0	7 ± 0	5 ± 0	3 ± 0

#### Effect of detergents

The desizing ability of amylase was checked in the presence of various detergents. TEGWA scale was used to evaluate the effects of wetting agents during fabric de-sizing. It was noticed that Tween 80 has no significant effect on the desizing potential of amylase, whereas Triton-X significantly increased the desizing of fabric. The combination of amylase and TritonX resulted in a high desizing of fabric at 60 °C (TEGWA scale 9) (Table 5).

**Table 5:** Desizing ability of amylase in the presence of amylase from *Bacillus cereus* AS2.

Temperature (°C)	Detergent				
	Amylase	Amylase + 0.5% Triton-X	0.5% Triton-X	Amylase + 0.5% Tween-80	0.5% Tween-80
30	2 ± 0	3 ± 0	2 ± 0	2 ± 0	2 ± 0
35	2 ± 0	3 ± 0	2 ± 0	2 ± 0	2 ± 0
40	3 ± 0	4 ± 0	2 ± 0	3 ± 0	2 ± 0
45	3 ± 0	4 ± 0	2 ± 0	3 ± 0	2 ± 0
50	5 ± 0	5 ± 0	2 ± 0	5 ± 0	2 ± 0
55	6 ± 0	6 ± 0	2 ± 0	6 ± 0	2 ± 0
60	6 ± 0	9 ± 0	2 ± 0	6 ± 0	2 ± 0
65	5 ± 0	8 ± 0	2 ± 0	5 ± 0	2 ± 0
70	3 ± 0	6 ± 0	2 ± 0	3 ± 0	2 ± 0

### Fabric desizing under optimized conditions

After biological and chemical desizing, results were compared with commercially desized fabric. The results showed that factory-desized and enzyme-desized fabrics have almost the same properties. That means, no significant changes in enzyme desized and acid desized fabrics were recorded. Water absorbance and weight loss for commercial and enzyme-treated fabric were also recorded same (Table 6). Reduction in tensile strength was observed in case of wrap and weft. As compare to acid treatment low reduction rate was observed when fabric was treated enzymatically. The reduction percent in wrap and weft was recorded as two and nine percent, respectively (Table 7).

**Table 6:** Fabric desizing under optimized conditions and comparison with a commercially desized fabric.

Samples	Tested parameters								
	Temperature (°C)	Time (h)	% weight loss	Initial length (cm)	Final length (cm)	Initial width (cm)	Final width(cm)	TEGEA rating	Wettability drop test (s)
Enzyme desized	60	15	8.9	25	24.5	15	14.7	9	9
5N HCl	RT	1	7.2	25	24.7	15	15	9	830
Factory desized	60	12	N/A	N/A	N/A	N/A	N/A	9	< 1
H20 (control)	60	15	2	25	25	15	15	2	150
Non-desized	N/A	N/A	N/A	N/A	N/A	N/A	N/A	2	1150

**Table 7:** Tensile strength of fabric (enzyme desized, acid desized and factory desized).

Sample	Wrap	Weft				
	Mean strength (LB)	Gain or loss (previous treatment)	Gain or loss (%)	Mean strength (LB)	Gain or loss (previous treatment)	Gain or loss (%)
*Bacillus cereus* AS2 desized	91.14	− 2.15	− 2.30	63.09	− 6.22	− 8.97
Acid desized	80.40	− 11.67	− 12.6	57.35	− 13.24	− 18.75
Factory desized	93.03	1.34	1.46	60.88	− 3.54	− 5.49
Control (d/w)	97.47	4.33	4.25	59.51	0.15	0.25
Non-desized	93.09	0	0	57.42	0	0

### Scanning electron micrograph of desized fabric

**Figure 3:**
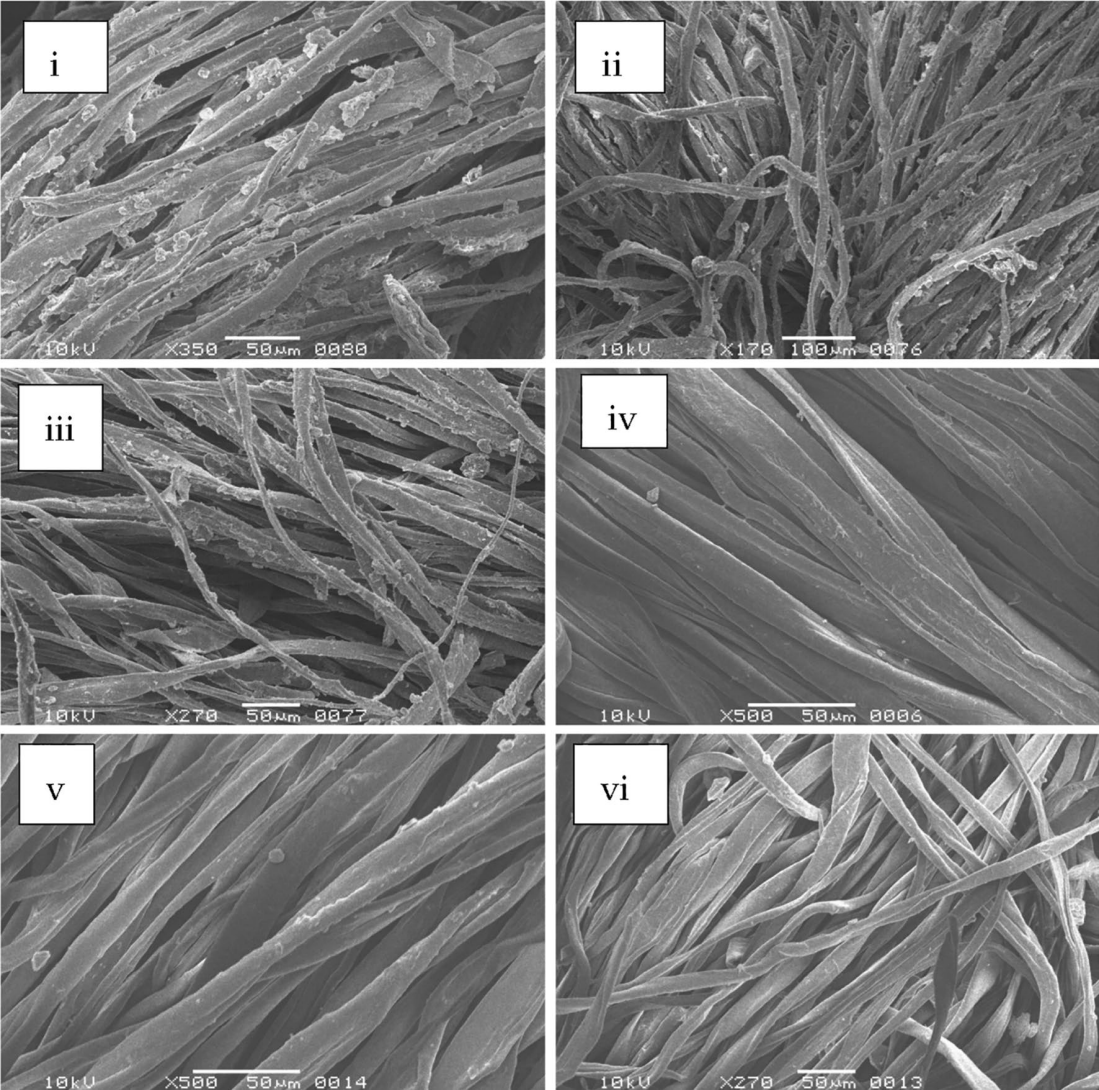
SEM results for fabric samples. (**i**) control (untreated sample), (**ii**) water treated, (**iii**) inactivated enzyme, (**iv**) commercially desized, (**v**) chemically desized, (**vi**) desizing using amylase from *Bacillus cereus* AS2.

SEM micrograph of the control, water, and inactivated enzyme-treated fabric showed that starch particles remain adhere to the fabric surface. No starch particle was detected in the case of enzyme, acid, and industrially desized fabric, whereas in the control multiple starch granules were found attached to the fibers. Moreover, no fabric damage was noticed in amylase-treated fabric samples (Fig. 3).

## Discussion

Desizing is a necessary process in the preparation of fabrics before dyeing and printing. The removal of small particles from fabric surfaces is critical. Previously, chemical treatments were used to desize fabric, but today eco-friendly materials such as enzymes are being employed as alternative desizing agents that are not only environmentally friendly but also human and machine friendly. Enzyme catalyze starch hydrolysis in both homogenous and heterogeneous systems, but the activities of enzymes in the heterogeneous system are different as these involve insoluble starch on the fabric surface. During desizing not only optimum properties of amylases are necessary but adsorption of enzymes to size particles on fabric surfaces is also necessary. The activity of the enzyme depends on enzyme structure, stability, surface area, size quantity of fabric surface, and transfer of enzyme from aqueous solution to the soil surface i.e. the fabric^25^.

Enzymes used in different textile sectors must be stable at various physiochemical conditions^23^. As per reports, scale 7–8 indicates 0–15% starch on warp threads and 0.075% starch on fabric whereas 0.08% and 0.04% starch on wrap and fabric (respectively) are indicated by scale 9^24^. In the current study, growth media with fabric samples was incubated at different periods. The enzyme synthesis was detected using a conventional enzyme assay after samples were taken at regular intervals. The highest amylase yield (1200 IU/ml/min) was seen after 72 h of incubation, and longer fermentation times were not determined to be adequate for enzyme production. The TEGEWA scale was used to determine the de-sizing potential (after fermentation). In SEM micrographs (during all incubation durations), starch particles were detected to adhere to the fabric surface but no fabric damage was seen. Fermentative de-sizing of cloth using a Bacillus strain that produces α-amylase has been previously reported^26^.

Fabric desizing using the traditional method, i.e., in the presence of HClwas investigated at different time intervals in a current tudy. Within 1 h, the fabric was desized to TEGEWA scale 9 in the presence of 5N HCl. Similarly, for effective fabric desizing, mineral acids such as sulphuric acid or hydrochloric acid were used at concentrations of 15 to 20 g/l (at room temperature). However, if temperature and drying are not properly controlled, this may tenderize the fabric, resulting in dyeing imperfections, fabric degradation, and loss of natural and smooth appearance. As a result, enzymatic desizing is currently the recommended method^27^

Desizing procedures are typically carried out at high temperatures ranging from 60 to 100 °C^26^. Pre-treatment of the fabric before the desizing stage consumed 50–60% of the process’s energy, therefore optimizing various process parameters can assist to reduce the process’s energy consumption^10,28^. Low temperature and short time are significant in terms of cost effectiveness, resistance to corrosion, less equipment degradation, and the necessity for enzyme stabilizers^26,28^. In current study physical conditions were optimized for fabric desizing. Maximum fabric desizing was noticed at 60 °C and 15 h. The optimum concentration of enzyme units for fabric de-sizing (TEGEWA scale 6) was found to be 1000 IU/ml at temperatures ranging from 55 to 60 °C, whereas higher enzyme concentrations were not found to have any significant impact on desizing might be because this enzyme concentration is optimal for the available substrate. Many studies have previously reported cloth desizing by improving amylase process parameters^28,29^. Haq, Ali^30^ optimized temperature, duration, pHand enzyme concentration for best desizing of grey fabric by *Bacillus amyloliquifaciens* mutant strains, reporting 100% desizing at 60 °C and 200–250 (IU/ml) amylase. Similarly, Saxena, Dutt^31^ observed fabric desizing at high temperatures (95 °C) using a partly isolated Bacillus specie SI-13 enzyme to (TEGEWA scale 9) in 20 min, but desizing (TEGEWA scale 7–8) took longer at low temperatures (50–70 °C) (40–60 min). Desizing through acid-stable amylase from Aspergillus was reported at low temperatures^32^.

Wetting agents are used in the coloration, preparation, and finishing of fabric. These substances improve the penetration of substances throughout the fiber of the fabric. Triton-X (detergent) showed improved desizing efficacy in the current study, whereas Tween-80 had no significant effect on desizing. Our findings are consistent with those of Battan, Dhiman^1^, who found that surfactants (Triton X-100 and Tween 20) play a role in the enzymatic desizing of cotton and grey cloth. Sadhasivam, Thanaraj^33^, and colleagues previously reported on the use of wetting agents during fermentative de-sizing.

The outcomes of commercially desized and enzyme desized fabric were compared after optimizing settings for maximal desizing. Wettability and weight reduction are crucial factors to consider when evaluating desizing effectiveness. Wettability and weight loss were found to be nearly the same in both enzymes desized and commercially desized fabric samples. When the weight loss of enzyme desized fabric was compared to the weight loss of water-desized fabric, a 22 percent efficiency in desizing was discovered demonstrating the efficacy of amylase in removing starch residues from the fabric surface. Kalpana, Sindhulakshmi^34^ previously reported a 31 percent weight reduction when amylase was producedusing a low-cost medium whereas 34.45 percent weight loss was oobserved by usingcommercially available amylase.

Hydrophobic impurities, dried starch film and other impurities in fabric yarn contribute to poor absorbency; however, our study found that a shorter absorption time (9 s) resulted in significant impurity elimination from the fiber surface. The lowest observed absorbency value for amylase desized fabric from *B. amyloliquefaciens* was 24 s in prior work^35^, demonstrating the importance of our technique. *Bacillus subtilis* S8-18 amylases was also found by Kalpana, Sindhulakshmi^34^ to have the shortest absorbency time (3 s). Moreover, reduction in tensile strength of fabric was less in case of enzymatic treatment whereas acid treatment resulted in a considerale high rate of reduction in tensile strength. These findings also strengthen the importance of bio-based desizing procedure at commercial level.

Furthermore, starch particles were detected adhering to the surface of untreated, water-treated, and inactivated enzyme-treated fabric. Whereas commercially available enzyme and acid desized fabrics were devoid of starch particles. In enzyme desized fabric, no surface damage was observed. Kalpana, Sindhulakshmi^34^ used SEM pictures to visualize desizing and discovered substantial evidence for *Bacillus subtilis* S818 enzyme in the textile industry. Battan, and Dhiman^1^ also used SEM micrographs to show enzymatic action during cloth de-sizing. Amylase from *Bacillus cereus* AS2 is attractive for eco-friendly textile desizing at moderate temperatures and for a short period and is one of the strong potential candidates for future applications in this field.

## Conclusions

Starch is applied to fabric surfaces in order to strengthen fabric string during weaving process. Desizing is a crucial step in fabric preparation before the printing and dyeing process. Sized particles are removed from fabric surfaces by using various chemicals such as diluted HCl and sulphuric acid. Chemical desizing not only results in damage to fabric surfaces but also caused adverse environmental pollution. Therefore, biological de-sizing is a preferred method nowadays. In the current study desizing potential of fabric was compared by using enzymes and chemicals. It was noticed that the fabric desized to TEGWA scale 9 within 72 h by using an amylase enzyme from *Bacillus cereus* AS2, whereas within 1 h complete de-sizing occurred in the presence of 5N HCl. Optimizing of desizing conditions showed that at a temperature range of 55–60 °C, the complete desizing was achieved within 15 h. 1000 IU/ml of the enzyme was found be the optimum concentration for maximum desizing of the tested fabric. The effect of detergents showed that Tween 80 does not affect fabric de-sizing whereas Triton-X desized fabric most significantly. Finally, the results of the enzyme desized fabric were compared to the factory desized sample, and it was observed that weight loss and water absorbency were almost the same that shows the potency of amylase from *Bacillus cereus* AS2 for fabric desizing.

## Data Availability

All data generated or analysed during this study are included in this published article.
